# Sonographic optic nerve sheath diameter as a surrogate measure for intracranial pressure in anesthetized patients in the Trendelenburg position

**DOI:** 10.1186/s12871-015-0025-9

**Published:** 2015-03-31

**Authors:** Ji-Hyun Chin, Hyungseok Seo, Eun-Ho Lee, Joohyun Lee, Jun Hyuk Hong, Jai-Hyun Hwang, Young-Kug Kim

**Affiliations:** 1 Department of Anesthesiology and Pain Medicine, Asan Medical Center, University of Ulsan College of Medicine, 88 Olympic-ro 43-gil, Songpa-gu, Seoul 138-736 Republic of Korea; 2 Department of Urology, Asan Medical Center, University of Ulsan College of Medicine, Seoul, South Korea

**Keywords:** Intracranial pressure, Optic nerve sheath diameter, Trendelenburg position

## Abstract

**Background:**

It remains to be elucidated whether the Trendelenburg position increases intracranial pressure (ICP). ICP can be evaluated by measuring the sonographic optic nerve sheath diameter (ONSD). We investigated the effect of the isolated Trendelenburg position on ONSD in patients undergoing robot-assisted laparoscopic radical prostatectomy. Additionally, we evaluated the effect of the Trendelenburg position combined with pneumoperitoneum on ONSD.

**Methods:**

Twenty-one patients scheduled for robot-assisted laparoscopic radical prostatectomy were enrolled. Sonographic ONSDs and hemodynamic parameters were measured at specific time points: in the supine position after induction of anesthesia, 3 min after the steep Trendelenburg position (35° incline), 3 min after the steep Trendelenburg position combined with pneumoperitoneum, and in the supine position after desufflation of the pneumoperitoneum.

**Results:**

The ONSD 3 min after the steep Trendelenburg position was significantly higher than that of the supine position after induction of anesthesia (5.1 ± 0.3 mm *vs.* 4.5 ± 0.4 mm). In addition, the ONSD 3 min after the steep Trendelenburg position combined with pneumoperitoneum was higher than that of the supine position after induction of anesthesia (4.9 ± 0.4 mm *vs.* 4.5 ± 0.4 mm). The ONSD in the supine position after desufflation of the pneumoperitoneum was similar to that in the supine position after induction of anesthesia.

**Conclusions:**

Use of the isolated steep Trendelenburg position, for even a short duration, increased the sonographic ONSD, providing a better understanding of the effect of only a transient steep Trendelenburg position on ONSD as a surrogate measure for ICP.

## Background

The Trendelenburg position has long been believed to increase intracranial pressure (ICP), an idea that may be derived from previous reports [1,2]. In these studies, the ICP increased when prolonged comatose patients with severe brain injury and neurosurgical patients were placed in the head-down position [1,2]. However, the Trendelenburg position differs from the head-down position used in those studies, in that the head is straight along the axis of the body in the Trendelenburg position, whereas the head is extended back and the body remains straight in the head-down position. In addition, the combined effect of a prolonged Trendelenburg position and pneumoperitoneum has been reported to increase ICP [3]. It is notable that the specific effect of the isolated Trendelenburg position on ICP has not been fully evaluated, given the frequent use of the Trendelenburg position in non-neurologic patients in daily practice. The invasiveness of the conventional method for measuring ICP might not allow the ICP of non-neurologic patients to be evaluated.

It has recently been determined that the ICP can be evaluated by noninvasive ocular sonography [4-7]. The retrobulbar segment of the optic nerve is surrounded by a distensible subarachnoid space, which can inflate when cerebrospinal fluid pressure increases [8,9]. An increased optic nerve sheath diameter (ONSD) detected by ocular sonography is associated with clinical signs of high ICP in adults with traumatic brain injury and in children with hydrocephalus or liver failure [9-13]. Moreover, the ONSD has been correlated with ICP invasively measured by an intraparenchymal catheter inserted into the frontal lobe in sedated patients receiving neurocritical care, including patients with severe traumatic brain injury [14]. Rapid and safe sonographic measurement of ONSD may enable patients to be screened for increased ICP.

We, therefore, aimed to investigate the effect of the isolated Trendelenburg position on ONSD to examine possible changes in ICP in patients undergoing robot-assisted laparoscopic radical prostatectomy. Additionally, we evaluated the effect of the Trendelenburg position combined with pneumoperitoneum on ONSD in these patients.

## Methods

### Patients

The study protocol was approved by the Institutional Review Board at Asan Medical Center (AMC IRB 2013-0408) and written informed consent was obtained from each patient scheduled for robot-assisted laparoscopic radical prostatectomy. This study was registered with the Clinical Research Information Service (KCT0000746). Patients with pre-existing neurological or cerebrovascular disease were excluded.

After routine hemodynamic monitoring (electrocardiogram, noninvasive blood pressure, and pulse oximetry) was applied and sensors for an INVOS 5100 cerebral/somatic oximeter (Coviden Inc., Dublin, Ireland) were attached to the right and left frontal areas, anesthesia was induced using a bolus intravenous injection of 5 mg/kg thiopental sodium followed by 0.1 mg/kg vecuronium. After tracheal intubation, patients were mechanically ventilated with a tidal volume of 8 ml/kg of ideal body weight at a respiratory rate of 10–16 breaths/min to maintain end-tidal CO_2_ partial pressure (ETCO_2_) between 30 and 35 mmHg. Positive end-expiratory pressure was not applied and the inspiratory-to-expiratory time ratio was set at 1:2. Anesthesia was maintained with 2–3 vol% sevoflurane plus a continuous infusion of 2–5 ng/ml remifentanil with 50% oxygen using medical air.

### Ocular sonography

The ONSD was measured sonographically by investigators trained in ocular sonography, as described previously [15,16]. Briefly, patients were placed in the supine position with their eyes closed, and a thick gel layer was applied to the closed upper eyelid. A 7.5-MHz linear probe was placed on the gel without excessive pressure and adjusted to the proper angle to display the optimal contrast between the retrobulbar echogenic fat tissue and the vertical hypoechoic band. An ultrasound beam was focused on the retrobulbar area using the lowest possible acoustic power that could measure the ONSD. The ONSD was measured at 3 mm behind the optic disc. Measurements were performed in the transverse and sagittal planes of both eyes, and the mean values of four measurements at each time point were used for analysis. To determine intra-observer and inter-observer variability, a random sample of 25% of ONSD measurements was submitted twice to the first investigator and once to a second investigator. The inter-observer variability was then calculated as the mean absolute difference between the two readings from the first and second investigators divided by their mean and expressed as a percentage. Similarly, the intra-observer variability was calculated as the mean absolute difference between the two readings from the first investigator divided by their mean and expressed as a percentage.

### Study protocol

When hemodynamically stable conditions were reached, four measurements were taken, as follows: in the supine position after induction of anesthesia (T_SUP_), 3 min after the steep Trendelenburg position (35° incline) (T_TREN_), 3 min after the steep Trendelenburg position combined with pneumoperitoneum (15 mmHg of insufflation pressure) (T_T+P_), and in the supine position after desufflation of the pneumoperitoneum (T_END_). After T_TREN_, the position was changed to the supine position for 10 min, which was followed by T_T+P_. Parameters measured at each time point included the ONSD, systolic blood pressure (SBP), diastolic blood pressure (DBP), mean blood pressure (MBP), heart rate (HR), tympanic body temperature (BT), airway peak pressure (P_peak_), airway plateau pressure (P_plat_), ETCO_2_, and regional cerebral oxygen saturation (rSO_2_) using near-infrared spectroscopy. In addition, arterial CO_2_ partial pressure (PaCO_2_), and hemoglobin concentration were measured at T_SUP_ and T_END_.

### Statistical analysis

Continuous variables are expressed as mean ± SD or median (interquartile range). Categorical variables are expressed as number (percentage). A power analysis based on our pilot study data suggested that a minimum sample size of 17 patients would be required to detect a mean 0.5 mm difference (SD: 0.58 mm) in the ONSD between T_SUP_ and T_TREN_ with a power of 90% at a significance of *P* < 0.05. Expecting a dropout rate of about 20%, we aimed to enroll 21 patients. One-way repeated measures analysis of variance (ANOVA) was used to evaluate the effect of several different positions under general anesthesia on the change in the ONSD, SBP, DBP, MBP, HR, P_peak_, P_plat_, and rSO_2_. If any interaction was significant, *post hoc* analysis was performed using the Holm–Sidak method. One-way repeated measures ANOVA on rank was used to evaluate the effect of the different positions under general anesthesia on the change in the BT and ETCO_2_. If any interaction was significant, *post hoc* analysis was performed using Tukey’s test. A paired *t* test was performed to evaluate PaCO_2_ and hemoglobin concentrations measured at T_SUP_ and T_END_. A *P* value < 0.05 was considered statistically significant. All statistical analyses were performed using SigmaPlot software, version 12.3 (Systat Software Inc., San Jose, CA).

## Results

Twenty-one patients scheduled for robot-assisted laparoscopic radical prostatectomy were enrolled and evaluated. All 21 enrolled patients completed the study protocol. The patients’ characteristics are shown in Table 1. Of the 21 patients, 12 had preoperative hypertension. There was no significant difference in MBP between patients with and without hypertension. There was no patient with chronic obstructive pulmonary disease.

**Table 1:** **Demographic and perioperative data**

Age (years)	63.6 ± 7.5
Height (cm)	167.1 ± 5.9
Weight (kg)	66.9 ± 7.3
Hypertension	12 (57.1%)
Diabetes mellitus	3 (14.3%)
Hemoglobin (g/dl)	
Immediately after induction	13.4 ± 0.9
At the end of surgery	11.9 ± 1.1
Operation time (min)	172.9 ± 33.3
Anesthetic time (min)	227.5 ± 38.9

Values are mean ± SD or number (percentage).

**Figure 1:**
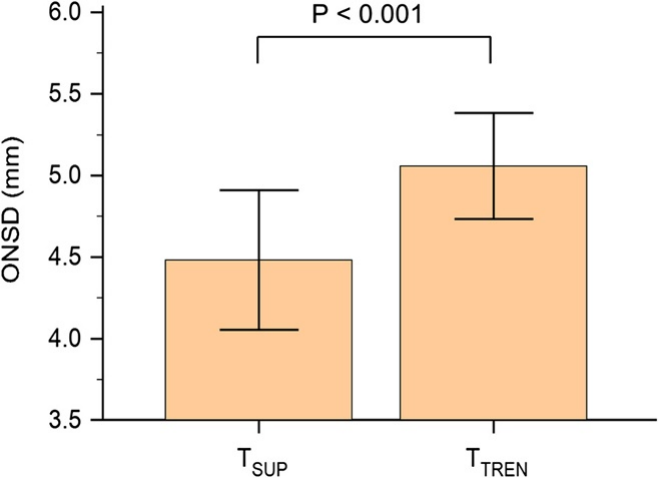
**Change in optic nerve sheath diameter (ONSD) between the supine and the steep Trendelenburg position.** A significant increase was found in the ONSD 3 min after the patient position was changed from supine to steep Trendelenburg. T_SUP_: in the supine position after induction of anesthesia; T_TREN_: 3 min after the steep Trendelenburg position (35° incline).

The ONSDs at several positions were significantly different from that of the supine position during surgery (*P* < 0.001) (Table 2). The ONSD at T_TREN_ significantly increased compared with that at T_SUP_ (5.1 ± 0.3 mm *vs.* 4.5 ± 0.4 mm; Figure 1). When patients with or without preoperative hypertension were separately considered, similar results were shown (with: 4.5 ± 0.5 mm at T_SUP_*vs.* 5.1 ± 0.3 mm at T_TREN_, *P* < 0.001; without: 4.5 ± 0.4 mm at T_SUP_*vs.* 5.0 ± 0.3 mm at T_TREN_, *P* = 0.003).

**Table 2:** **Intraoperative variables**

	T_SUP_	T_TREN_	T_T+P_	T_END_
ONSD (mm)	4.5 ± 0.4	5.1 ± 0.3^*^	4.9 ± 0.4^*^	4.4 ± 0.4
SBP (mmHg)	106.9 ± 20.2	99.6 ± 14.2	132.7 ± 19.8^*^	112.5 ± 16.6
DBP (mmHg)	56.8 ± 10.5	56.0 ± 7.0	76.7 ± 10.8^*^	61.7 ± 13.0
MBP (mmHg)	73.5 ± 13.1	70.5 ± 8.9	95.3 ± 11.7^*^	78.6 ± 13.8
HR (beats/min)	66.4 ± 9.6	59.2 ± 8.5	60.2 ± 7.5	65.3 ± 9.1
BT (°C)	36.5 (36.3–36.8)	36.3 (36.0–36.7)	36.1 (35.8–36.3)^*^	35.9 (35.5–36.5)^*^
P_peak_ (cmH_2_O)	12.1 ± 2.2	18.1 ± 3.1^*^	26.8 ± 4.0^*^	15.5 ± 2.4^*^
P_plat_ (cmH_2_O)	11.5 ± 2.1	17.5 ± 3.1^*^	25.9 ± 4.0^*^	14.3 ± 2.5
ETCO_2_ (mmHg)	32.0 (31.0–33.0)	30.0 (29.5–32.0)^*^	33.0 (32.0–34.0)	35.0 (32.5–36.5)^*^
PaCO_2_ (mmHg)	40.3 ± 3.0	NA	NA	47.1 ± 4.0^*^
rSO_2_ (%)	73.6 ± 5.5	70.3 ± 4.6	70.9 ± 5.3	72.8 ± 3.8

Values are mean ± SD or median (interquartile range). ONSD: optic nerve sheath diameter; SBP: systolic blood pressure; DBP: diastolic blood pressure; MBP: mean blood pressure; HR: heart rate; BT: tympanic body temperature; P_peak_: airway peak pressure; P_plat_: airway plateau pressure; ETCO_2_: end-tidal CO_2_ partial pressure; PaCO_2_: arterial CO_2_ partial pressure; rSO_2_: regional cerebral oxygen saturation by near-infrared spectroscopy; NA: not available; T_SUP_: in the supine position after induction of anesthesia; T_TREN_: 3 min after the steep Trendelenburg position (35° incline); T_T+P_: 3 min after the steep Trendelenburg position combined with pneumoperitoneum; T_END_: in the supine position after desufflation of the pneumoperitoneum. * P < 0.05 compared with T_SUP_.

In addition, the ONSD at T_T+P_ increased compared with that at T_SUP_. At T_END_, the ONSD was similar to that at T_SUP_. The intra- and inter-observer variabilities of the ONSD measurement were 1.8% and 2.8%, respectively. Intraoperative variables, including hemodynamic and respiratory variables, at each time point are shown in Table 2. The rSO_2_ did not show significant changes across all time points.

## Discussion

We found in our present study that the ONSD, as measured by ocular sonography, increased 3 min after the isolated steep Trendelenburg position in mechanically ventilated patients. In addition, we observed that the ONSD increased after the patient was placed in the steep Trendelenburg position combined with pneumoperitoneum.

The ONSD measured by ocular sonography has been found to correlate with ICP, suggesting that it could be used as a surrogate for ICP. Various cut-off values have been used to distinguish high from normal ICP [6,7,13-15]. Several previous studies reported that the ONSD cut-off value of 5 mm was sensitive and specific for the identification of computed tomographic findings suggestive of increased ICP [6,13]. Importantly, the ONSD cut-off value of 5 mm was found to be able to distinguish an ICP > 20 mmHg measured by ventriculostomy, showing an area under a receiver operator characteristics curve of 0.93 with a sensitivity of 88% and specificity of 93% in patients with clinical or radiologic signs of increased ICP [14]. On the other hand, a previous study used several lower cut-off values, from 4.7 mm to 4.9 mm, as well as 5.0 mm, to investigate the accuracy of each cut-off in detecting high ICP in patients with fluctuating and stable ICP [17].

Physicians often establish the Trendelenburg position in both surgical and non-surgical conditions, such as when implementing central venous catheterization to facilitate venous access, performing surgical procedures on the lower abdominal or pelvic organs to obtain a good surgical field, and treating hypotensive patients to improve hemodynamics, despite a lack of evidence for its effects. However, the position has been considered to increase ICP, which has a normal range of 7–15 mmHg in supine healthy adults [18]. This increase was seen in previous studies that found that the head-down position increased ICP in neurologic patients [1]. However, the head-down position, which is frequently performed during neurologic surgery, is different from the Trendelenburg position. The head-down position extends the head and neck from the normal supine position, whereas the Trendelenburg position inclines the whole body in a supine position. In addition, few reports have included non-neurologic patients when evaluating the effect of the isolated Trendelenburg position on ICP. Therefore, we believe that our study design for assessing the effects of the position on ONSD as a surrogate measure for ICP is unique.

We found from our current analysis that the ONSD increased to approximately 5.0 mm immediately after patients were placed in the isolated steep Trendelenburg position, reflecting a high ICP, defined as 20 mmHg [14]. The Trendelenburg position may disturb cerebral venous drainage by increasing the impedance of the lungs to inflation [19]. Therefore, when the lungs are ventilated with the same tidal volume used for the supine position, the Trendelenburg position may lead to higher intrathoracic pressure and subsequently increase the ICP by the transmission of intrathoracic pressure to the intracranial space [20]. Indeed, our current results indicated increased airway pressure during the Trendelenburg position. It has been reported that the ONSD increased after desufflating pneumoperitoneum and before resuming a neutral level at the end of surgery [21]. This previous finding might support our results. However, the PaCO_2_ is expected to increase at the end of surgery, although this increase was not shown in the previous study, and this increased PaCO_2_ might affect the ONSD. Therefore, the effect of the isolated Trendelenburg position on ONSD may not have been exclusively evaluated in the previous study.

In contrast to our current results, it has been reported that the ONSD measured 10 min after pneumoperitoneum combined with the Trendelenburg position did not increase under general anesthesia with sevoflurane [22]. Increased cerebral blood flow might be compensated for by cerebrospinal fluid translocation to the vascular component during the 10 min interval between the position change and the measurement of the ONSD. However, our present study measured the ONSD 3 min after pneumoperitoneum combined with the Trendelenburg position. This duration might be too short for the patient to develop compensation mechanisms. Interestingly, another study assessed the ONSD during laparoscopic prostatectomy, and found an increased ONSD 10 min after pneumoperitoneum combined with the Trendelenburg position [3]. This previous study used desflurane, which induced a greater increase in cerebral blood flow than sevoflurane [23], for anesthetic maintenance. Differences in the anesthetic drug might contribute, at least in part, to the conflicting results.

The effect of ETCO_2_ on ICP did not accentuate the increase in the ONSD observed in the steep Trendelenburg position because the ETCO_2_ level decreased during study period. Our present result suggests the importance of careful control of ETCO_2_ in anesthetized patients in the Trendelenburg position. However, the previous report might be confusing for clinicians when adjusting the ETCO_2_ during robot-assisted laparoscopic radical prostatectomy, because it showed an unexplained small difference between the ETCO_2_ and the PaCO_2_ over the whole period when the Trendelenburg position combined with pneumoperitoneum was adopted [3], unlike the results of our present analyses and previous studies [24-26].

We found that the rSO_2_ was unchanged at all of the predetermined time points under general anesthesia. The rSO_2_, which reflects a regional balance between cerebral oxygen supply and demand, has been studied to identify the effects of various positions on cerebral blood flow under diverse positions during laparoscopic surgery, yielding conflicting results [27-30]. The inconsistencies among studies may be partly due to the characteristics of near-infrared spectroscopy, which mainly reflects the venous compartment (artery:vein = 25%:75%), and its lack of accuracy for reflecting cerebral blood flow compared with jugular bulb oxygen saturation [31].

Our study has the following limitations. First, we studied a fixed degree of incline when establishing the Trendelenburg position. Further analysis of whether there is a graded association between the degree of incline during the Trendelenburg position and the change in the ONSD is needed. Second, our investigations were conducted under general anesthesia without excluding the effect of anesthetics on ONSD. Therefore, the change in the ONSD when the position was changed under general anesthesia can differ from that in awake patients, who were previously found not to show significant changes in the ONSD when placed in the Trendelenburg position [32]. An attenuated ability to maintain homeostasis in cerebral blood flow during general anesthesia using volatile anesthetics would be a plausible explanation, because volatile anesthetics have a dose-dependent cerebral vasodilatory effect [33]. Third, there are no general consensus over optimal cut-off values of ONSD, despite of use of ONSD cut-off value of 5 mm to detect increased ICP > 20 mmHg in the present study [6,7,13-15]. Furthermore, the accuracy of ONSD in detecting elevated ICP remains controversial [7,17,34]. Therefore, the present study does not definitively prove that there is an elevated ICP with steep Trendelenburg positioning, but does indicate that there is a likely rise in ICP which may reach critical levels in some patients.

## Conclusions

The sonographic ONSD as a surrogate measure for ICP increases when patients are placed in the isolated steep Trendelenburg position even for a short duration. Monitoring of the sonographic ONSD may provide useful information on changes in ICP when the steep Trendelenburg position is inevitably performed.

## Abbreviations

ICP: Intracranial pressure
ONSD: Optic nerve sheath diameter
ETCO_2_: End-tidal CO_2_ partial pressure
rSO_2_: Regional cerebral oxygen saturation
PaCO_2_: Arterial CO_2_ partial pressure
ANOVA: Analysis of variance
